# Comparing the Salt Tolerance of Different Spring Soybean Varieties at the Germination Stage

**DOI:** 10.3390/plants12152789

**Published:** 2023-07-27

**Authors:** Xinyu Zhou, Yumei Tian, Zhipeng Qu, Jinxing Wang, Dezhi Han, Shoukun Dong

**Affiliations:** 1 Agricultural College, Northeast Agricultural University, Harbin 150030, China; 2 Suihua Branch of Heilongjiang Academy of Agricultural Sciences, Suihua 152052, China; 3 Heihe Branch of Heilongjiang Academy of Agricultural Sciences, Heihe 164399, China

**Keywords:** spring soybeans, salt stress, germination, evaluation

## Abstract

Salinization is a global agricultural problem with many negative effects on crops, including delaying germination, inhibiting growth, and reducing crop yield and quality. This study compared the salt tolerance of 20 soybean varieties at the germination stage to identify soybean germplasm with a high salt tolerance. Germination tests were conducted in Petri dishes containing 0, 50, 100, 150, and 200 mmol L^−1^ NaCl. Each Petri dish contained 20 soybean seeds, and each treatment was repeated five times. The indicators of germination potential, germination rate, hypocotyl length, and radicle length were measured. The salt tolerance of 20 soybean varieties was graded, and the theoretical identification concentration was determined by cluster analysis, the membership function method, one-way analysis of variance, and quadratic equation analysis. The relative germination rate, relative germination potential, relative root length, and relative bud length of the 20 soybean germplasms decreased when the salt concentration was >50 mmol L^−1^, compared with that of the Ctrl. The half-lethal salt concentration of soybean was 164.50 mmol L^−1^, and the coefficient of variation was 18.90%. Twenty soybean varieties were divided into three salt tolerance levels following cluster analysis: Dongnong 254, Heike 123, Heike 58, Heihe 49, and Heike 68 were salt-tolerant varieties, and Xihai 2, Suinong 94, Kenfeng 16, and Heinong 84 were salt-sensitive varieties, respectively. This study identified suitable soybean varieties for planting in areas severely affected by salt and provided materials for screening and extracting parents or genes to breed salt-tolerant varieties in areas where direct planting is impossible. It assists crop breeding at the molecular level to cope with increasingly serious salt stress.

## 1. Introduction

Soybean (*Glycine max*) originated in China and was domesticated from wild soybean (*Glycine soja*) approximately 6000 to 9000 years ago [1]. It has become one of the world’s most widely cultivated and utilized economic and food crops, contributing to approximately 25% of the global edible oil market and approximately two-thirds of the world’s protein demand [2,3]. China is one of the largest soybean consumers in the world, and its import volume increases every year; therefore, it has an urgent need to increase its domestic soybean production [4]. Salinity is one of the main forms of abiotic stress and one of the most common agricultural problems in arid and semi-arid regions [5,6]. Salinity monitoring agencies such as the United States Salinity Laboratory (USSL) found that approximately 1 billion hectares of land out of the 13 billion hectares in the world are affected by salt, according to data from the Food and Agriculture Organization (FAO) of the United Nations [7]. Salt reduces the utilization rate of arable land and seriously affects plant growth and development depending on the plant growth stage, variety, and salinity level [8,9]. Therefore, identifying and screening soybean varieties suitable for growth under salt stress is of great ecological and economic significance. Soybeans have different sensitivities to salt stress at different growth stages [10]. Seed germination and post-germination growth are critical stages in plant growth and development, and salt tolerance at the germination stage plays a vital role in its survival and growth under salt conditions. Some morphological indicators of seeds at the germination stage are convenient and fast to measure, such as germination rate, germination potential, and bud length. This can effectively shorten the identification cycle and better reflect the true salt tolerance level of soybeans. Therefore, this stage is widely used to evaluate salt tolerance [11,12]. Germplasm resources with superior traits such as high yield, disease and insect resistance, and abiotic stress tolerance are the vital foundation for breeding programs. [13]. Cao et al. [14] evaluated the salt tolerance of 51 soybean germplasms from Indonesia using a hydroponic method and analyzed six varieties with enhanced salt tolerance. They revealed that the expression level of the salt tolerance gene Ncl was significantly positively correlated with salt tolerance. Sun et al. [15] performed a comprehensive evaluation of the salt-alkali tolerance of 21 rice varieties at the bud and seedling stages and identified four varieties with elevated salt-alkali tolerance and seven varieties with reduced salt-alkali tolerance. They also observed significant differences in the SKC1 and DST genes among varieties with different types of salt-alkali tolerance. Therefore, screening for salt-tolerant germplasm not only provides a basis for finding suitable salt-tolerant varieties for cultivation but also furnishes materials for molecular breeding and mining of salt-tolerant genes. This study determined the effects of different NaCl concentrations on the germination of 20 soybean germplasms from Heilongjiang Province. It aimed to solve the response rules of different soybean germplasms to salt stress at the germination stage and clarify their salt tolerance variation degree. Principal component analysis, weighted membership function, quadratic regression, and other statistical methods were used to analyze different indicators of each germplasm, and combined with the membership function mean values and the half-lethal concentrations of each indicator under different concentrations, the salt tolerance of each variety was clustered and appraised. Finally, based on the integration of the two clustering results, outstanding germplasms with high salt tolerance at the germination stage and appropriate identification concentrations were selected and provided materials for soybean salt tolerance breeding.

## 2. Results

### 2.1. Effects of Different Concentrations of NaCl on Various Indicators of Soybean Germination Stage

Salt tolerance identification at the germination stage was performed on 20 soybean varieties from different regions of Heilongjiang Province using NaCl solutions of different concentrations (Table 1). All indicators showed a gradually decreasing trend with the gradual increase in NaCl concentration. The decline of each indicator was more obvious when the NaCl concentration was above 100 mmol L^−1^.

Compared with that of the control, the vigor index (VI) and radicle length (RL) significantly decreased at 50 mmol^−1^ NaCl. Meanwhile, the changes in other indicators were not evident. This indicated that soybean seeds had a certain tolerance to low-concentration salt stress. However, the effects on various soybean indicators were significant at 100, 150, and 200 mmol L^−1^: the germination rate (GR) of soybean seeds decreased by 4.20%, 10.23%, and 30.37%, respectively, and the germination energy (GE) decreased by 11.65%, 25.35%, and 45.56%, respectively. Meanwhile, the mean values of the germination and vigor indices GI and VI decreased by 2.19, 3.98, and 6.02%; and 817.52, 1016.58, and 1171.79, respectively, compared to 20.14%, 36.61%, and 55.38%, and 63.21%, 78.61%, and 90.61% of the control group, respectively. The mean HL and RL decreased by 16.02 mm, 19.75 mm, and 24.94 mm, and 46.28 mm, 57.03 mm, and 67.79 mm, respectively, equivalent to 43.47%, 53.60%, 67.68%, and 57.89%, 71.34%, and 84.80% of the control group, respectively. This indicated that the salt environment has a significant inhibitory effect on the indicators at the germination stage, and the higher the concentration, the more evident the inhibitory effect. Among them, the VI, HL, and RL decreased the most with increasing salt concentration. This indicated that HL and RL are the most sensitive indicators reflecting the degree of salt’s effect on seed germination.

Salt stress has various effects on soybean seed germination, and different traits have varied sensitivities to salt stress. The coefficient of variation of each indicator of the test materials was not the same under different salt concentrations. This generally shows that 200 mmol L^−1^ > 150 mmol L^−1^ > 100 mmol L^−1^ > 50 mmol L^−1^ > Ctrl, except for the GR at 50 mmol L^−1^. The coefficients of variation of other indicators under the four salt treatments were all greater than 10%. This indicated that salt stress increased the difference between varieties, which is conducive to the comparison and screening of salt tolerance.

### 2.2. Salt Tolerance Coefficient of Each Single Indicator

Each single indicator of the different soybean varieties decreased compared with the control under NaCl treatment and further decreased with increasing NaCl concentrations (Supplementary Table S1). There were only significant differences in relative vigor index (RVI) and relative radicle length (RRL) under 50 mmol L^−^^1^ NaCl after synthesizing the salt tolerance coefficients of each variety (Table 1). Therefore, 50 mmol L^−^^1^ NaCl could not be used to distinguish the salt tolerance of the soybean germination stage. The salt tolerance of the soybean germination stage was comprehensively evaluated using 100, 150, and 200 mmol L^−1^ NaCl. The range and order of variation of the different indicators among the varieties were different (Supplementary Table S1). The use of a single indicator to evaluate the salt tolerance of soybean germplasm is usually limited and inaccurate.

### 2.3. Analysis of Variance (ANOVA) and Correlation Analysis of Each Single Indicator

The independent and interactive effects of two factors—different varieties and different NaCl concentrations—on the measured indicators were analyzed using a multifactor ANOVA. The sig values of the corresponding F values under different varieties and different NaCl concentrations were all below 0.01 for each indicator (Table 2). This indicated that the different varieties and NaCl concentrations had significant effects on the measured indicators. In addition, a significant interaction effect was observed between the variety and NaCl concentration, and the sig values of their F-values were all below 0.01. According to Pearson’s correlation coefficient analysis, there were different degrees of correlation between the salt tolerance coefficients of each indicator at the germination stage of the different soybean varieties under different NaCl concentrations (Table 3).

At 100 mmol L^−1^, there was a significant positive correlation between relative germination rate (RGR), relative germination energy (RGE), and relative germination index (RGI) (*p* < 0.01), and a weak positive correlation with RVI (*p* < 0.05); there was a significant positive correlation between RVI, RGI, and RRL (*p* < 0.01), and a significant positive correlation between relative hypocotyl length (RHL) and RVI (*p* < 0.01). At 150 mmol L^−1^, there was a weak positive correlation between RGR, RGE, and RGI (*p* < 0.05), a significant positive correlation between RGI, RGE, and RVI (*p* < 0.01), and a weak negative correlation between RHL and RRL (*p* < 0.05). At 200 mmol L^−1^, there was a significant positive correlation between RGR, RGE, and RGI (*p* < 0.01) and a weak negative correlation with RGI (*p* < 0.05); there was a weak positive correlation between RVI, RGI, and RHL (*p* < 0.05); and there was a weak positive correlation between RRL and RGR (*p* < 0.05).

These results showed that different varieties and NaCl concentrations had significant effects on various indicators of soybean seed germination. Furthermore, there was a significant interaction effect between them and a certain internal relationship between the salt tolerance indicators of different soybean varieties under different NaCl concentrations; these relationships changed under different NaCl concentrations. These findings can help us understand the mutual influence of different indicators and provide valuable information for breeding salt-tolerant soybean varieties. The information provided by different indicators has crossover and overlap. Therefore, it is necessary to perform a dimensionality reduction analysis on the indicators.

### 2.4. Principal Component Analysis of the Salt Tolerance Coefficient

Principal component analysis of the salt tolerance coefficients of RGR, RGE, RGI, RVI, RHL, and RRL (the six indicators of the soybean germination stage under different NaCl) (Table 4) extracted three principal components with contribution rates greater than 10%. Their cumulative contribution rates reached 86.177%, 93.695%, and 80.383% at 100, 150, and 200 mmol L^−1^ NaCl, respectively, which represented most of the information for the six traits.

The eigenvalue of the first principal component was 3.06 at 100 mmol L^−1^ NaCl. This accounted for 50.992% of the variance. The most influential trait was RVI (eigenvalue of 0.861). This indicated that RVI was the most important indicator in the first principal component. Meanwhile, RGI, RVI, and RGE had the highest eigenvalues. The eigenvalues of the second and third principal components were 1.22 and 0.892, respectively, accounting for 20.326% and 14.859% of the variance. The most influential traits were RHL for both components.

The eigenvalue of the first principal component was 2.635 at 150 mmol L^−1^ NaCl. This accounted for 43.916% of the variance, which was lower than that at 100 mmol L^−1^ NaCl. At this concentration, RGI became the most important indicator (eigenvalue of 0.884). Among them, RGE, RGI, and RVI had the highest eigenvalues. The eigenvalues of the second and third principal components were 1.181 and 1.007, respectively, accounting for 19.685% and 16.781% of the variance. The most influential traits were RHL and RRL, respectively.

The eigenvalue of the first principal component was 3.739 at 200 mmol L^−1^ NaCl. This accounted for 62.316% of the variance. The RGI remained the most important indicator (eigenvalue of 0.977). RGE, RGI, and RVI had the highest contributions. The eigenvalues of the second and third principal components were 1.056 and 0.827, respectively, accounting for 17.598% and 13.78% of the variance. The most influential traits were RHL and RRL.

### 2.5. Comprehensive Evaluation of Salt Tolerance

The weights of each indicator were calculated according to the contribution rates of the comprehensive indicators F1, F2, and F3 under different NaCl. This indicated the relative importance of each principal component under the corresponding stress. Simultaneously, the comprehensive evaluation values of salt tolerance (D values) of different soybean varieties under different NaCl were calculated according to the membership function values and indicator weights of each comprehensive indicator in different materials (Table 5).

The weights of the three comprehensive indicators were 59.17%, 23.59%, and 17.24%, respectively, under 100 mmol L^−1^ NaCl. The D values of 20 varieties ranged from 0.19 to 0.89; Jiadou 18 had the largest D value, indicating that it had the best comprehensive performance of salt tolerance at this concentration, followed by Heihe 49 and Heike 58, whose D values were all above 0.8; and Suinong 94 had a small D value, indicating that it was more sensitive to this concentration. Meanwhile, the weights of the three comprehensive indicators under 150 mmol L^−1^ NaCl were 54.63%, 24.49%, and 20.88%, respectively. The D values of 20 varieties ranged from 0.21 to 0.83; Heike 58 had the largest D value. This indicated that it had the best comprehensive performance in salt tolerance at this concentration, followed by Hefeng 50, whose D value was above 0.7. Meanwhile, Kenfeng 16, Heinong 84, and Xihai 2 had small D values, indicating that they were more sensitive to this concentration. The weights of the three comprehensive indicators under 200 mmol L^−1^ were 66.51%, 18.78%, and 14.71%, respectively. The D values of the 20 varieties ranged from 0.01 to 0.86; 10 had the largest D value, indicating that it had the best comprehensive performance of salt tolerance at this concentration, followed by Heihe 49 and Dongnong 253, whose D values were above 0.7. Meanwhile, Heinong 84, Kenfeng 16, and Suinong 94 had small D values, indicating that they were more sensitive to this concentration.

The D value rankings differ because different NaCls have variable effects and modes on soybeans, resulting in unique adaptability and resistance in different soybean varieties. In addition, it was necessary to combine the indicators under different salt concentrations since the weights of the comprehensive indicators were variable under different concentrations.

### 2.6. Regression Analysis

We first simplified the NaCl concentrations of Ctrl, 50, 100, 150, and 200 mmol L^−1^ to 0, 1, 2, 3, and 4, respectively. We then performed one-way quadratic regression analysis on the relative values of each indicator and salt concentration to obtain the equation and its coefficients (Supplementary Table S2). All varieties with a negative coefficient showed a trend toward promoting their growth under low-concentration NaCl, according to the characteristics of the one-way quadratic equation. The RGR, RGE, and RGI indicators particularly showed that over half of the varieties had a negative coefficient value of X2. This indicated that the promotional effect of low NaCl on seed germination was most obvious. In addition, varieties 4, 7, and 8 were more sensitive to salt stress at the initial stage of germination according to the RGV indicator, and their absolute values of coefficient a were larger than those of other varieties. The absolute values of coefficient a for varieties 7 and 12 decreased on the seventh day. This indicated that they showed better recovery and adaptability under continuous salt stress.

A more accurate screening concentration was obtained by predicting the half-lethal concentration of each indicator according to the LC_50_ formula combined with the coefficients listed in Supplementary Table S2; the results are summarized in Table 6. The mean value of the salt tolerance half-lethal concentration of different soybean varieties was 3.29, which was 164.50 mmol L^−1^, and the coefficient of variation was 18.90%. The mean values of LC_50_ under RVI, RHL, and RRL were relatively low (1.61, 2.67, and 1.63, respectively, which were 80.50, 133.50, and 81.50 mmol L^−1^, respectively). This showed that these indicators were more sensitive to salt stress, and their coefficients of variation were 19.60–29.00%, while the LC_50_ of RGR, RGE, and RGI were relatively large, with mean values of 5.57, 4.46, and 3.81, respectively, which were 278.50, 223.00, and 190.50 mmol L^−1^, respectively, and their coefficients of variation were 24.50–34.20%. In general, there were large differences between the LC_50_ values of the different salt tolerance indicators.

### 2.7. Cluster Analysis of Salt Tolerance

Salt tolerance is the adaptation of plants to salt stress and is a comprehensive manifestation of various metabolic processes. A comprehensive evaluation of the salt tolerance of plants at the germination stage should consider the seed’s germination ability under salt stress and the normal growth of seedlings after germination. The employment of a single NaCl concentration cannot objectively and truly reflect the salt tolerance of soybean germplasm; there were large differences in the strength of salt tolerance among the different varieties under different NaCl concentrations (Table 5).

**Figure 1:**
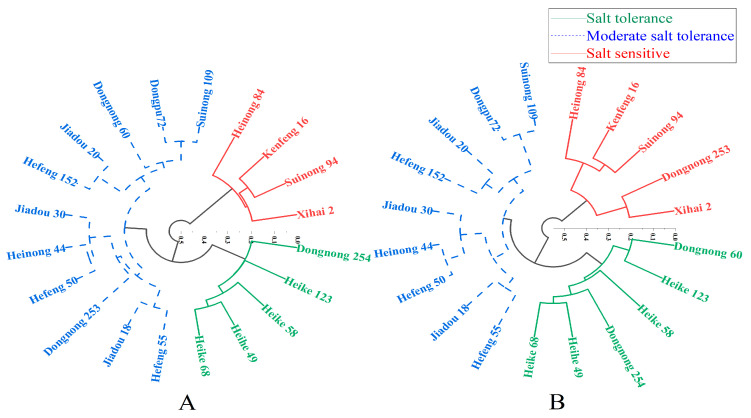
Cluster analysis of the salt tolerance of 20 soybean varieties under NaCl stress. Note: (**A**) Cluster analysis under all salt concentration stresses; (**B**) LC_50_ cluster analysis. Hierarchical cluster analysis was performed using D value and Origin; the coordinate axis represents Euclidean distance.

Therefore, based on the average values of the membership function values of RGR, RGE, RGI, RVI, RHL, and RRL under different concentrations of salt stress, this study conducted a cluster analysis on the salt tolerance of twenty soybean germplasms. At a Euclidean distance of 0.3, twenty soybean germplasms were divided into three groups (Figure 1A). Figure 1A showed that varieties Dongnong 254, Heike 123, Heike 58, Heihe 49, and Heike 68 had strong salt tolerance and are salt-tolerant varieties; Hefeng 55, Jiadou 18, Dongnong 253, Hefeng 50, Henong 44, Jiadou 30, Hefeng 152, Jiadou 20, Dongnong 60, Dongpu 72, and Suinong 109 had moderate salt tolerance; however, Xihai 2, Suinong 94, Kenfeng 16, and Heinong 84 are salt-sensitive varieties.

A half-lethal concentration (LC_50_) of 3.30 obtained from the analysis was brought into the regression equation to obtain the relative values of various indicators at the semi-lethal concentration. Then use this relative value for membership function analysis and the obtained membership function value for cluster analysis to obtain Figure 1B. Twenty soybean germplasms were divided into three groups at a Euclidean distance of 0.35: Dongnong 60, Heike 123, Heike 58, Heihe 49, Heike 68, and Dongnong 254 had strong salt tolerance and are salt-tolerant varieties; Hefeng 55, Jiadou 18, Hefeng 50, Heinong 44, Jiadou 30, Hefeng 152, Jiadou 20, Dongpu 72, and Suinong 109 had moderate salt tolerance; Xihai 2, Dongnong 253, Suinong 94, Kenfeng 16, and Heinong 84 are salt-sensitive varieties.

A synthesis of the two clustering methods concluded that varieties Dongnong 254, Heike 123, Heike 58, Heihe 49, and Heike 68 were salt-tolerant, while varieties Xihai 2, Suinong 94, Kenfeng 16, and Heinong 84 were salt-sensitive varieties. Photographs of representative seedlings from each variety were taken after 7 days of treatment with control, 100 mmol L^−1^ NaCl, and 150 mmol L^−1^ NaCl (Supplementary Table S3) to illustrate the phenotypic differences among different soybean varieties under salt stress.

## 3. Discussion

Salt stress inhibits plant growth, and the rate of growth reduction depends on various factors, such as plant species, developmental stage, and salt concentration [16]. Slow development is an adaptive mechanism of plants to survive adverse conditions; this can reduce water consumption, salt absorption, and damage caused by salt stress [17]. The seed germination stage is the initial stage of plant growth and development; it is one of the most sensitive stages to salt stress [18]. Na^+^ and Cl^−^ ions cause lipid peroxidation of the seed coat cell membrane, increased mechanical resistance, and decreased permeability of the seed coat, thereby inhibiting water infiltration into the cotyledon and hypocotyl and affecting seed germination [19,20]. Meanwhile, the replacement of Ca^2+^ with Na^+^ ions to bind to cell wall polysaccharides destroys cell wall elasticity and stability, and Cl^−^ decomposes cell wall components, resulting in damaged cell integrity [21,22]. Salt stress also causes metabolic disorders in cells, inhibits DNA replication and transcription, reduces RNA polymerase activity in the nucleus, and inhibits protein synthesis and amylase activity. This affects physiological processes such as cell division and differentiation, glycogen hydrolysis, and respiration [23,24,25,26]. Salt stress also severely affects the osmotic balance in cells, resulting in decreased osmotic potential and increased water potential. This inhibits water absorption, turgor pressure, and elongation of hypocotyl cells and affects hypocotyl growth and emergence [27]. Salt-tolerant varieties can exclude or isolate Na^+^ and Cl^−^ ions to reduce the salt concentration in cells while increasing the absorption and accumulation of K^+^ to maintain the ion balance between inside and outside cells; however, sensitive varieties cannot [28,29,30]. In addition, salt-tolerant varieties increase the synthesis and accumulation of osmotic regulators, such as proline and soluble sugar, to improve the intracellular/extracellular osmotic balance, thereby reducing the impact of salt stress on cells [31]. This study showed that Dongnong 60, Hefeng 55, and Heihe 49 varieties showed a promoting effect on GR and germination potential at 50 mmol L^−1^ NaCl. This is because low salt concentrations can stimulate seed water absorption and metabolism and promote seed germination. However, the RGR, RGE, RRL, and RHL of different soybean varieties decreased to varying degrees above 50 mmol L^−1^ NaCl. This indicated that high salt concentrations exceeded the salt tolerance threshold of most soybean germplasm. This inhibits seed water absorption under the dual effects of high osmotic potential and ion toxicity, leading to a decrease in various indicators, which also reflects a difference in salt tolerance between different soybean varieties.

Plant salt tolerance is a complex process influenced by a combination of biochemical and molecular mechanisms [32]. Therefore, a single indicator cannot comprehensively evaluate salt tolerance. The comprehensive evaluation method considers the correlation between various indicators and the difference in importance between different indicators. This provides a more scientific and accurate reflection of the resistance of different varieties [33]. This study performed ANOVA and correlation analyses on the salt tolerance coefficients of various indicators at different concentrations. There was a significant interaction effect between different varieties and NaCl concentrations, and there was a very significant or significant correlation between multiple indicators at different concentrations. The principal component analysis results of soybean germplasm under different NaCl concentrations were not completely consistent. The rankings of membership functions in the different germplasms also differed. Therefore, a cluster analysis was performed on the salt tolerance of the 20 soybean germplasms based on the average membership function values of various indicators under different NaCl concentrations. They were divided into three categories: salt-tolerant germplasms (Dongnong 254, Heike 58, Heike 123, and 5 other varieties); salt-sensitive germplasms (Xihai 2, Suinong 94, Kenfeng 16, and Heinong 84); and moderately salt-tolerant germplasms (Dongnong 60, Jiadou 18, Dongnong 253, and 11 other varieties).

A selection of the germplasm salt tolerance identification concentration should ensure that the test indicators show significant stress traits compared with the control and confirm that they do not result in the test materials coming close to death to cover up the real salt tolerance situation [34]. The half-lethal concentration (LC_50_) is often used as a screening method for plant stress concentrations [35]. The mean half-lethal salt concentration in this study was 3.29 (Supplementary Table S2 and Table 6). The theoretically optimal NaCl concentration to identify soybean salt tolerance was 164.50 mmol L^−1^. This indicated that there is a maximum difference between the different varieties at this concentration. Shi et al. [36] studied the effects of 150 mmolL^−1^ NaCl on soybean germination. In addition, Ravelombola et al. [37] found that 150 mmol L^−1^ NaCl is a reasonable concentration to determine cowpea salt tolerance. This study performed a cluster analysis on the salt tolerance of 20 soybean germplasms based on the membership function values of various indicators under half-lethal salt stress. They were divided into three categories: salt-tolerant germplasms (Jiadou 20, Heike 58, Heike 123, and 6 other varieties); salt-sensitive germplasms (Xihai 2, Suinong 94, Dongnong 253, and 5 other varieties); and moderately salt-tolerant germplasms (Jiadou 18, Hefeng 55, Hefeng 50, and 9 other varieties).

Simultaneously, using the membership function values of various indicators under half-lethal salt stress and comparing them with the average membership function values of various indicators under different NaCl concentrations, a comprehensive evaluation of salt tolerance was performed. The results show that the screening results of salt-tolerant and salt-sensitive germplasms in 20 soybean germplasms basically agree, but there are still some differences. This may be due to the different abilities of various soybean germplasms to resist salt stress. Some germplasm lines had stronger salt tolerance under low salt stress, whereas others had stronger salt tolerance under high salt stress. However, screening for salt-tolerant germplasm with the average values of various indicators under different salt stresses may cause mutual cancellation of salt tolerance information before and after salt stress.

This study only screened for germplasm at the soybean germination stage. It is also necessary to evaluate and identify the salt tolerance at the seedling stage for the salt-sensitive germplasm and the salt-tolerant germplasm and determine their salt tolerance thresholds to ensure the accuracy and reliability of the screened materials. The varieties that are stable during both periods can be used as research materials for studying the molecular mechanisms of soybean salt tolerance, laying the foundation for studying soybean salt tolerance mechanisms and breeding salt-tolerant varieties.

## 4. Materials and Methods

### 4.1. Materials

The 20 soybean varieties tested were all from Heilongjiang Province, China (121°10′–135°5′ E, 43°25′–53°33′ N) (Table 7).

### 4.2. Experimental Design

Seeds with uniform shape and size (full grains) were disinfected with 0.1% KMnO4 solution for 10 min, rinsed with deionized water 3–5 times, soaked in deionized water for 2 h, the surface moisture was absorbed, and they were placed in a culture dish (diameter, 11 cm) with two layers of filter paper laid in advance. Twenty grains were placed in each culture dish, and NaCl stress solutions (50, 100, 150, and 200 mmol L^−1^) were administered to saturation. Five replicates were used for each concentration, and deionized water was used as the Ctrl. The culture dishes were placed in an artificial climate chamber and cultured at 20 °C/25 °C (13 h dark/11 h light). The filter paper and solution in the culture dish were replaced daily, and the salt solution was kept sufficiently stable. The number of germinated seeds (the radicle protruding by 2 mm was taken as the germination criterion) was counted daily from the second day onward, and other morphological indicators were measured on the seventh day of treatment.

### 4.3. Measured Indicators

The GI of each variety was calculated by counting the number of germinated seeds every day during the experiment. The GE and GR of each variety were calculated on the 3rd and 7th days of cultivation, respectively. Ten germinated seeds were randomly selected from each replicate, and their radicle and HL were measured using a vernier caliper. The seedling growth amount (radicle + HL) was multiplied by the GI to obtain the VI [38,39]. The measured results of the above indicators were compared with those of the control group. The control group consisted of seeds cultivated under salt-free conditions. The relative values of each indicator (the salt tolerance coefficient) were calculated by dividing the measured results of each indicator under salt stress conditions by the corresponding measured results of the control group. The calculation formulas are shown below (See Supplementary Table S4 for the meaning of the acronyms):

GE = number of germinated seeds in 3 days/number of seeds tested(1)

RGE (%) = (treatment germination energy/control germination energy) ×100(2)

GR = number of germinated seeds in 7 days/number of seeds tested(3)

RGR (%) = (treatment germination rate/control germination rate) ×100%(4)

RHL (%) = (treatment hypocotyl length/control hypocotyl length ×100%(5)

RRL (%) = (treatment radicle length/control radicle length) ×100(6)

GI = ∑ (Gt/Dt)(7)

Gt refers to the number of germinated seeds within time t days, and Dt is the corresponding germination days(8)

RGI (%) = (treatment germination index/control germination index) ×100(9)

VI = S × GI (GI: germination index, S: seedling growth)(10)

RVI (%) = (treatment vigor index/control vigor index) ×100(11)

### 4.4. Data Statistics and Analysis

The data were organized in Microsoft Excel 2016 and analyzed via principal component analysis, Pearson correlation analysis, analysis of variance, membership function analysis, and quadratic regression analysis using IBM SPSS 23.0 statistical software to comprehensively identify the salt tolerance of the test materials. Hierarchical cluster grouping and data plotting were performed using Origin 2021.

The correlation between indicators was analyzed according to the salt tolerance coefficient of each indicator, and possible information crossover and overlap were found. Principal component analysis was performed on the salt tolerance coefficients under the same NaCl concentration to reduce the dimensionality of the variables and extract the first P principal components (comprehensive indicators) with cumulative contribution rates greater than 85% to replace the original salt tolerance coefficients for soybean salt tolerance evaluation. The values of the different materials for the first P principal components were calculated, and the membership function method was used to standardize the data. The comprehensive evaluation index D value was used as the basis for the ranking. The larger the D value, the better the comprehensive traits. The membership formula is as follows:

(12) $$\mu \left(X_{j}\right)=\frac{X_{j}-X_{min}}{X_{max}-X_{min}}j=1,2,3,\ldots ,n$$

where *μ*(*X_j_*) represents the value of germplasm *j* (*j* = 1, 2, ……, 20) after standardization on the *p*th principal component (comprehensive indicator); *X_j_* represents the value of the *j*th germplasm on the *p*th principal component; *X_max_* and *X_min_* are the maximum and minimum values of all germplasm on the *p*th principal component, respectively [40]. The weights of each comprehensive indicator are calculated according to the size of the principal component contribution rate:

(13) $$\omega p=\frac{\varphi p}{\sum_{p=1}^{n}\varphi p},p=1,2,3,\ldots ,n$$

where *ωp* represents the weight of the *p*th comprehensive indicator among all comprehensive indicators, *φ p* is the contribution rate of the *p*th comprehensive indicator [41]. Comprehensive evaluation index *D* values.

(14) $$D\sum_{i}^{n}\left[U\left(x_{i}\right)\times W_{i}\right],i=1,2,\ldots ,n$$

The average value was calculated, and cluster analysis was performed using this average value after obtaining the membership function values of each indicator at different salt concentrations [42].

A one-way quadratic regression analysis was performed on the relative values of each test indicator and salt concentration using SPSS23: Y = ax^2^ + bx + c (where a, b, and c are equation coefficients). Calculate the value of x when each relative index is 0.5 (Y = 0.5) according to the obtained quadratic regression equation, that is, the salt concentration under half-lethal conditions (LC_50_), and perform cluster analysis on the obtained LC_50_ values to determine the salt tolerance level of different varieties under half-lethal conditions. The arithmetic mean of the LC_50_ values was calculated to determine the concentration. The LC_50_ mean value was substituted into a one-way quadratic equation to obtain the relative values of each indicator under the identification concentration condition. Membership function analysis was performed on this relative value, and cluster analysis was performed on the obtained membership function values [43].

The rationality of the calculated identification concentration was determined by synthesizing the results of the two types of cluster analyses to evaluate the salt tolerance levels of the different varieties more comprehensively.

## 5. Conclusions

This study evaluated the salt tolerance of different soybean varieties at the germination stage and screened out the varieties that are suitable for saline-alkali land growth. Increasing NaCl concentrations had a significant inhibitory effect on soybean seed germination, and the optimal NaCl concentration for soybean salt tolerance screening was 164.50 mmol L^−1^, according to regression analysis. Comprehensive comparison by membership function and cluster analysis revealed that Dongnong 254, Heike 123, Heike 58, Heihe 49, and Heike 68 are salt-tolerant varieties, while Xihai 2, Suinong 94, Kenfeng 16, and Heinong 84 are salt-sensitive varieties.

## Figures and Tables

**Table 1:** Comparison of indices under salt stress in the soybean germination stage.

Treatment	Index	GR (%)	GE (%)	GI	VI	HL (mm)	RL (mm)
Ctrl	Max	98.00	98.0	15.68	2275.28	52.33	120.32
	Min	82.00	70.00	7.11	653.03	20.75	52.34
	Average	91.35	87.15	10.87	1293.25	36.85	79.94
	SE	0.05	0.08	2.792	507.712	9.68	20.71
	CV	0.047	0.09	0.26	0.39	0.21	0.26
50 mmol L^−1^	Max	98.00	98.00	18.05	2064.30	54.49	107.48
	Min	77.00	55.00	5.82	292.29	16.40	22.74
	Average	90.50	81.95	10.43	906.02	31.19	49.79
	SE	0.063	0.132	3.55	542.54	0.06	0.06
	CV (%)	0.07	0.161	0.34	0.60	0.26	0.39
100 mmol L^−1^	Max	100.00	100.00	14.93	1046.10	31.37	63.76
	Min	65.00	34.00	4.31	185.75	11.32	20.90
	Average	87.15	77.00	8.68	475.73	20.83	33.66
	SE	0.092	0.166	2.87	224.73	6.00	11.56
	CV	0.106	0.216	0.33	0.47	0.26	0.34
150 mmol L^−1^	Max	98.00	93.00	10.55	524.51	23.64	41.24
	Min	62.00	41.00	3.97	23.61	9.23	8.95
	Average	82.00	65.05	6.89	276.67	17.10	22.91
	SE	0.119	0.15	2.04	128.45	3.65	7.99
	CV	0.145	0.24	0.30	0.47	0.29	0.35
200 mmol L^−1^	Max	94.00	92.00	9.58	251.69	17.48	25.11
	Max	15.00	9.00	1.02	14.92	5.49	5.50
	Average	63.60	47.45	4.85	121.46	11.91	12.15
	SE	0.217	0.237	2.32	63.39	2.90	5.41
	CV	0.34	0.5	0.48	0.52	0.24	0.45

Note: This experimental data is based on the results of 20 soybean varieties under different salt treatments. The values are the Max, Average, SE, and CV of all varieties (five replicates) at each concentration. Ctrl, control; CV, coefficient of variation; GE, germination energy; GI, germination index; GR, germination rate; HL, hypocotyl length; RL, radicle length; SE, standard error; VI, vigor index.

**Table 2:** Multifactor analysis of variance.

Source	(RGR)		(RGE)		(RGI)		(RVI)		(RHL)		(RRL)	
	F	Sig.	F	Sig.	F	Sig.	F	Sig.	F	Sig.	F	Sig.
Variety	12.065	<0.01	13.549	<0.01	165.494	<0.01	36.585	<0.01	26.252	<0.01	3.208	<0.01
NaCl concentration	150.563	<0.01	111.255	<0.01	58.747	<0.01	1000.623	<0.01	813.131	<0.01	216.183	<0.01
Variety and NaCl concentration	4.887	<0.01	2.772	<0.01	161.683	<0.01	23.314	<0.01	5.726	<0.01	1.942	<0.01

Note: ANOVA. The F-test was used for the analysis of variance. The F value in the result represents a specific value obtained by the F test formula, and the corresponding *p*-value is obtained according to the numerical table, that is, sig. A signal (sig) value <0.05 indicates an influence on the result; otherwise, there is no influence. RGE, relative germination energy; RGI, relative germination index; RGR, relative germination rate; RHL, relative hypocotyl length; RRL, relative radicle length; RVI, relative vigor index. The table below is the same.

**Table 3:** Correlation analysis.

Indicator	100 mmol L^−1^					
	RGR	RGE	RGI	RVI	RHL	RRL
RGR	1					
RGV	0.587 **	1				
RGI	038 **	0.512 **	1			
RVI	0.304 **	0.447 **	0.75 **	1		
RHL	−0.083	0.184	−0.014	0.30 **	1	
RRL	0.062	0.058	0.106	0.359 **	0.3 **	1
Indicator	150 mmol L^−1^					
	RGR	RGE	RGI	RVI	RHL	RRL
RGR	1					
RGV	0.27 *	1				
RGI	0.2 *	0.659 **	1			
RVI	0.172	0.422 **	0.766 **	1		
RHL	−0.068	0.042	−0.105	−0.15	1	
RRL	0.085	0.173	0.275 *	0.177	−0.239 *	1
Indicator	200 mmol L^−1^					
	RGR	RGE	RGI	RVI	RHL	RRL
RGR	1					
RGV	0474 **	1				
RGI	−0.285 *	0.337 **	1			
RVI	0.444 **	0.52 **	0.238 *	1		
RHL	−0.228 *	0.009	−0.206 *	0.227 *	1	
RRL	0.201 *	0.123	−0.038	0.2 *	−0.086	1

Note: Correlation Analysis of Indexes of 20 Soybean Varieties Using the Pearson Correlation Coefficient Method: ** indicates a significant correlation at the level of 0.01 (double-tailed); * indicates a significant correlation at the level of 0.05 (double-tailed).

**Table 4:** Principal component analysis table.

Determination of Indicators	100 mmol L^−1^			150 mmol L^−1^			200 mmol L^−1^		
	F1	F2	F3	F1	F2	F3	F1	F2	F3
RGR	0.765	−0.49	0.246	0.584	0.636	−0.098	0.85	0.03	−0.474
RGE	0.828	−0.264	0.396	0.738	0.402	−0.19	0.893	−0.105	−0.302
RGI	0.833	−0.03	−0.17	0.884	−0.003	0.375	0.977	0.07	−0.029
RVI	0.861	0.349	−0.167	0.759	−0.402	0.425	0.896	0.099	0.287
RHL	0.061	0.759	0.641	−0.441	0.652	0.286	0.095	0.979	0.13
RRL	0.592	0.458	−0.455	0.443	−0.166	−0.747	0.673	−0.268	0.641
Eigenvalue	3.06	1.22	0.892	2.635	1.181	1.007	3.739	1.056	0.827
Variance contribution rate/%	50.992	20.326	14.859	43.916	19.685	16.781	62.316	17.598	13.78
Cumulative contribution rate/%	50.992	71.318	86.177	43.916	63.601	80.383	62.316	79.914	93.695

Note: Using principal component analysis, F1, F2, and F3 are the principal component values corresponding to each trait.

**Table 5:** Comprehensive evaluation of salt tolerance.

Variety	100 mmol L^−1^					150 mmol L^−1^					200 mmol L^−1^				
	μ(F1)	μ(F2)	μ(F3)	D	Sort	μ(F1)	μ(F2)	μ(F3)	D	Sort	μ(F1)	μ(F2)	μ(F3)	D	Sort
Xihai 2	0.31	0.61	0.06	0.34	18	0.32	0.25	0.27	0.29	18	0.23	0.36	0.76	0.33	16
Suinong 94	0.00	0.68	0.15	0.19	20	0.24	0.38	0.59	0.35	17	0.06	0.06	0.31	0.10	18
Suinong 109	0.34	0.16	0.39	0.30	19	0.42	0.69	0.23	0.45	13	0.46	0.46	0.30	0.43	12
Kenfeng 16	0.62	0.55	0.67	0.61	7	0.00	0.67	0.24	0.21	20	0.00	0.00	0.25	0.04	19
Jiadou 30	0.60	0.38	0.70	0.56	11	0.44	0.85	0.56	0.57	8	0.55	0.55	1.00	0.61	5
Jiadou 20	0.39	0.66	1.00	0.56	12	0.37	0.90	0.70	0.57	7	0.43	0.43	0.38	0.42	13
Jiadou 18	0.99	1.00	0.38	0.89	1	0.51	0.36	0.42	0.45	12	0.33	0.33	0.13	0.30	17
Heinong 84	0.71	0.32	0.53	0.59	9	0.23	0.21	0.48	0.28	19	0.00	0.00	0.09	0.01	20
Heinong 44	0.51	0.31	0.24	0.42	17	0.46	0.77	1.00	0.65	4	0.63	0.63	0.44	0.60	7
Heike 68	1.00	0.48	0.28	0.75	4	0.82	0.46	0.08	0.58	6	1.00	1.00	0.08	0.86	1
Heike 58	0.90	0.62	0.68	0.80	3	1.00	0.71	0.52	0.83	1	0.69	0.69	0.12	0.61	6
Heike 123	0.99	0.38	0.32	0.73	5	0.59	0.26	0.19	0.43	14	0.66	0.66	0.04	0.57	8
Heihe 49	1.00	0.49	0.54	0.80	2	0.67	0.71	0.08	0.56	9	0.84	0.84	0.24	0.76	2
Hefeng 55	0.86	0.31	0.00	0.58	10	0.43	0.16	0.45	0.37	16	0.50	0.50	0.31	0.47	11
Hefeng 50	0.57	0.68	0.76	0.63	6	0.67	0.76	0.95	0.75	2	0.52	0.52	0.26	0.48	9
Hefeng 152	0.50	0.64	0.86	0.60	8	0.34	1.00	0.41	0.52	10	0.47	0.47	0.46	0.47	10
Dongnong 60	0.54	0.11	0.57	0.44	16	0.20	0.88	0.28	0.38	15	0.40	0.40	0.29	0.38	14
Dongpu72	0.52	0.15	0.68	0.46	15	0.69	0.55	0.83	0.68	3	0.42	0.42	0.13	0.37	15
Dongnong 253	0.40	0.73	0.39	0.48	14	0.57	0.00	0.86	0.49	11	0.71	0.71	0.69	0.71	3
Dongnong 254	0.69	0.00	0.68	0.52	13	0.78	0.72	0.00	0.60	5	0.74	0.74	0.00	0.63	4
Weight coefficient/%	59.17	23.59	17.24			54.63	24.49	20.88			66.51	18.78	14.71		

Note: Using the membership function method, μ(F1), μ(F2), and μ(F3) are the membership function values corresponding to each soybean variety.

**Table 6:** Salt-tolerant half-lethal concentration of soybean.

Variety	Half Lethal Concentration (LC_50_)						Average
	RGR	RGE	RGI	RVI	RHL	RRL	
Xihai 2	4.17	2.80	4.02	1.30	1.23	1.38	2.48
Suinong 94	3.61	2.60	1.91	1.32	2.96	1.57	2.33
Suinong 109	8.14	4.38	3.43	1.28	2.61	1.84	3.61
Kenfeng 16	3.43	3.30	2.74	1.25	3.11	1.63	2.58
Jiadou 30	6.18	4.03	4.04	1.40	3.41	1.43	3.42
Jiadou 20	6.21	4.69	3.26	1.10	3.40	1.33	3.33
Jiadou 18	4.26	3.48	3.50	2.21	2.96	2.21	3.10
Heinong 84	3.47	3.43	3.17	1.50	2.33	1.42	2.55
Heinong 44	5.29	3.36	4.12	0.95	1.67	1.21	2.77
Heike 68	7.83	8.68	4.77	2.99	2.50	2.42	4.87
Heike 58	5.28	4.51	4.20	1.70	2.87	1.59	3.36
Heike 123	5.32	4.67	3.90	2.16	2.20	1.88	3.35
Heihe 49	7.88	8.13	4.16	2.29	2.69	1.72	4.48
Hefeng 55	4.55	3.39	3.72	1.61	1.92	2.11	2.88
Hefeng 50	5.74	4.00	4.03	1.54	3.22	1.34	3.31
Hefeng 152	6.09	4.73	3.32	1.53	4.13	1.67	3.58
Dongnong 60	5.91	4.39	3.08	1.33	2.67	1.68	3.18
Dongpu72	5.35	4.53	3.65	1.45	2.36	1.23	3.09
Dongnong 253	6.10	4.01	6.92	1.49	2.86	1.39	3.80
Dongnong 254	6.51	6.07	4.25	1.88	2.31	1.58	3.77
Average	5.57	4.46	3.81	1.61	2.67	1.63	3.29
CV (%)	24.50%	34.20%	24.80%	29.00%	23.90%	19.60%	18.90%

Note: The values are the half-lethal salt concentrations x when each indicator is 0.5 (Y = 0.5), obtained from the quadratic function (Y = ax^2^ + bx + c) in Supplementary Table S2.

**Table 7:** Information on 20 soybean varieties.

No	Code	Cultivars	Source
I	1	Xihai 2	Xinhai Soybean Cooperative in Binxian County, Heilongjiang Province
II	2	Suinong 94	Suihua Branch of Heilongjiang Academy of Agricultural Sciences
	3	Suinong 109	
III	4	Kenfeng 16	Heilongjiang Academy of Land Reclamation Sciences
IV	5	Jiadou 30	Jiamusi Branch of Heilongjiang Academy of Agricultural Sciences, Heilongjiang Guangmin Seed Industry Co., Ltd.
	6	Jiadou 20	
	7	Jiadou 18	
V	8	Heinong 84	Soybean Research Institute of Heilongjiang Academy of Agricultural Sciences
	9	Heinong 44	
VI	10	Heike 68	Heihe Branch of Heilongjiang Academy of Agricultural Sciences
	11	Heike 58	
	12	Heike 123	
	13	Heihe 49	
VII	14	Hefeng 55	Jiamusi Branch of Heilongjiang Academy of Agricultural Sciences
	15	Hefeng 50	
	16	Hefeng 152	
VIII	17	Dongnong 60	Soybean Research Institute of Northeast Agricultural University
	18	Dongpu72	
	19	Dongnong 253	
	20	Dongnong 254	

## Data Availability

Not applicable.

## Supplementary Materials

The following supporting information can be downloaded at: https://www.mdpi.com/article/10.3390/plants12152789/s1, Supplementary Table S1: Salt tolerance coefficient of each index; Supplementary Table S2: Coefficient table of quadratic regression equation in one variable; Supplementary Table S3: Photos of different soybean varieties under salt stress during germination; Supplementary Table S4: Acronym list.
