# Supplementary material for: Comparing the Salt Tolerance of Different Spring Soybean Varieties at the Germination Stage
Source: Plants (Basel). 2023 Jul 27;12(15):2789. doi: 10.3390/plants12152789 (PMC10421394; doi:10.3390/plants12152789)
Supplement: Supplementary file 1 [file plants-12-02789-s001.zip › plants-2500254-supplementary.pdf]

Supplementary Table S1. Salt tolerance coefficient of each index.

Variety	Treatment	Salt tolerance coefficient					
		(RGR)	(RGE)	(RGI)	(RVI)	(RHL)	(RRL)
Xihai 2	50	0.97	0.85	0.9	0.52	0.63	0.49
	100	0.8	0.63	0.74	0.35	0.5	0.41
	150	0.84	0.53	0.53	0.2	0.43	0.31
	200	0.49	0.11	0.33	0.08	0.34	0.14
Suinong 94	50	1	0.77	0.75	0.64	1.07	0.65
	100	0.76	0.48	0.51	0.24	0.54	0.37
	150	0.74	0.62	0.54	0.2	0.5	0.26
	200	0.36	0.23	0.25	0.04	0.31	0.08
Suinong 109	50	1	0.94	0.97	0.68	0.96	0.73
	100	0.95	0.83	0.52	0.19	0.44	0.41
	150	0.95	0.69	0.56	0.19	0.51	0.32
	200	0.87	0.54	0.48	0.07	0.31	0.1
Kenfeng 16	50	1	0.97	0.82	0.56	0.94	0.63
	100	0.97	0.91	0.72	0.35	0.64	0.45
	150	0.76	0.63	0.42	0.03	0.51	0.25
	200	0.16	0.16	0.1	0.02	0.34	0.17
Jiadou 30	50	0.99	0.98	0.97	0.63	0.97	0.57
	100	0.97	0.89	0.84	0.34	0.6	0.35
	150	0.91	0.72	0.71	0.18	0.58	0.29
	200	0.81	0.51	0.51	0.11	0.55	0.12
Jiadou 20	50	0.97	0.96	0.88	0.47	0.9	0.5
	100	0.93	0.86	0.57	0.24	0.75	0.38
	150	0.85	0.83	0.63	0.18	0.58	0.24
	200	0.77	0.61	0.38	0.06	0.36	0.13
Jiadou 18	50	1.01	0.94	0.88	0.73	0.86	0.67
	100	0.98	0.93	0.85	0.62	0.71	0.64

	150	0.85	0.69	0.63	0.26	0.46	0.31
	200	0.57	0.24	0.35	0.08	0.28	0.17
Heinong 84	50	0.92	0.91	0.9	0.6	0.76	0.55
	100	0.97	0.96	0.89	0.41	0.53	0.38
	150	0.67	0.67	0.53	0.17	0.43	0.25
	200	0.26	0.23	0.16	0.03	0.25	0.1
Heinong 44	50	0.99	0.73	0.77	0.3	0.5	0.44
	100	0.96	0.71	0.92	0.27	0.49	0.41
	150	0.99	0.63	0.67	0.23	0.48	0.18
	200	0.74	0.56	0.51	0.12	0.39	0.18
Heike 68	50	1.01	1.02	1.05	1.95	0.93	0.92
	100	1	1.01	1.09	0.48	0.54	0.54
	150	0.94	0.96	0.76	0.23	0.36	0.34
	200	0.97	0.94	0.73	0.19	0.27	0.21
Heike 58	50	0.96	0.98	1.14	0.48	0.82	0.59
	100	0.99	0.98	0.99	0.54	0.67	0.42
	150	1.05	0.93	0.95	0.32	0.47	0.34
	200	0.74	0.62	0.56	0.13	0.29	0.21
Heike 123	50	1.01	1.07	1.1	0.92	0.76	0.6
	100	1.01	1.1	0.95	0.49	0.5	0.54
	150	0.82	0.83	0.62	0.24	0.38	0.32
	200	0.76	0.73	0.51	0.12	0.26	0.19
Heihe 49	50	0.98	0.97	1.11	0.95	0.79	0.62
	100	1.02	1.02	1.06	0.55	0.6	0.45
	150	0.95	0.95	0.63	0.22	0.46	0.34
	200	0.91	0.91	0.62	0.13	0.34	0.21
Hefeng 55	50	0.94	0.88	0.86	0.55	0.73	0.69
	100	1.05	0.84	0.88	0.48	0.42	0.55
	150	0.85	0.51	0.62	0.26	0.43	0.31
	200	0.63	0.37	0.44	0.12	0.34	0.18

Hefeng 50	50	1.03	0.88	0.91	0.58	0.97	0.52
	100	0.91	0.86	0.81	0.4	0.7	0.37
	150	0.98	0.77	0.83	0.29	0.53	0.25
	200	0.77	0.47	0.46	0.11	0.31	0.15
Hefeng 152	50	0.99	1	1	0.78	0.99	0.65
	100	0.96	0.86	0.65	0.29	0.72	0.42
	150	0.92	0.79	0.54	0.17	0.61	0.29
	200	0.8	0.6	0.4	0.07	0.39	0.14
Dongnong 60	50	1.07	1.02	1	0.71	0.94	0.7
	100	0.99	0.98	0.67	0.24	0.47	0.39
	150	1.02	0.6	0.43	0.11	0.51	0.26
	200	0.84	0.63	0.37	0.06	0.31	0.09
Dongpu72	50	0.97	0.89	0.9	0.59	0.87	0.52
	100	1	0.89	0.68	0.34	0.51	0.3
	150	1	0.84	0.73	0.28	0.38	0.21
	200	0.76	0.57	0.39	0.08	0.26	0.11
Dongnong 253	50	0.89	0.81	0.82	0.58	0.8	0.51
	100	0.81	0.74	0.81	0.35	0.62	0.4
	150	0.72	0.63	0.75	0.35	0.44	0.28
	200	0.64	0.56	0.64	0.16	0.46	0.17
Dongnong 254	50	1.1	1.05	1.16	0.92	0.88	0.76
	100	1.04	0.96	0.92	0.36	0.48	0.27
	150	1.09	0.91	0.68	0.21	0.39	0.34
	200	0.94	0.82	0.62	0.09	0.25	0.19

Note: The salt tolerance coefficient was calculated using the relative indicator method, and the five independent replicate samples of each variety under their respective treatments were calculated. RGE, relative germination energy; RGI, relative germination index; RGR, relative germination rate; RHL, relative hypocotyl length; RRL, relative radicle length; RVI, relative vigor index. The table below is the same.

Supplementary Table S2. Coefficient table of quadratic regression equation in one variable.

Variety	RGR			RGE			RGI			RVI			RHL			RRL		
	a	b	c	a	b	c	a	b	c	a	b	c	a	b	c	a	b	c
Xihai 2	-0.03	0.01	0.99	-0.03	-0.09	0.98	-0.01	-0.10	1.06	0.05	-0.43	0.97	-0.04	-0.33	0.97	0.05	-0.38	0.94
Suinong 94	-0.04	0.00	1.00	0.01	-0.20	0.97	-0.01	-0.23	0.98	0.05	-0.45	1.00	0.00	-0.19	1.07	0.04	-0.37	0.99
Suinong 109	-0.01	0.00	1.00	-0.01	-0.06	1.04	0.03	-0.26	1.05	0.06	-0.49	1.02	0.02	-0.26	1.05	0.02	-0.32	1.00
Kenfeng 16	-0.10	0.20	0.96	-0.08	0.11	0.98	-0.03	-0.08	0.98	0.05	-0.46	1.00	0.00	-0.16	1.03	0.04	-0.36	0.99
Jiadou 30	-0.02	0.02	1.00	-0.03	0.01	1.00	-0.02	-0.03	1.01	0.05	-0.43	1.00	0.25	0.23	1.05	0.05	-0.40	0.97
Jiadou 20	-0.01	-0.02	1.00	-0.02	-0.01	0.99	0.01	-0.18	1.01	0.07	-0.50	0.97	-0.02	-0.09	1.00	0.05	-0.42	0.96
Jiadou 18	-0.05	0.09	0.99	-0.07	0.12	0.97	-0.04	-0.01	0.98	-0.01	-0.21	0.99	-0.01	-0.13	1.00	0.01	-0.22	0.97
Heinong 84	-0.07	0.12	0.97	-0.07	0.12	0.96	-0.06	0.05	0.98	0.03	-0.37	0.98	0.02	-0.25	1.00	0.05	-0.39	0.97
Heinong 44	-0.03	0.07	0.98	0.03	-0.19	0.96	-0.02	-0.03	0.95	0.08	-0.52	0.92	0.06	-0.36	0.94	0.07	-0.45	0.95
Heike 68	0.00	-0.01	1.01	-0.01	0.02	1.00	-0.04	0.07	1.02	-0.05	-0.12	1.33	0.01	-0.25	1.05	0.01	-0.24	1.05
Heike 58	-0.04	0.10	0.96	-0.04	0.10	0.97	-0.07	0.16	1.01	0.03	-0.30	0.93	0.00	-0.16	1.00	0.05	-0.37	0.97
Heike 123	-0.02	0.03	1.01	-0.05	0.10	1.01	-0.04	0.03	1.04	0.01	-0.27	1.06	0.03	-0.29	1.01	0.03	-0.30	0.96
Heihe 49	-0.01	0.02	0.99	-0.01	0.02	1.00	-0.04	0.05	1.04	0.00	-0.24	1.06	0.02	-0.23	1.00	0.04	-0.35	0.98
Hefeng 55	-0.04	0.10	0.97	-0.02	-0.07	1.00	-0.03	-0.03	0.98	0.03	-0.34	0.96	0.05	-0.36	1.01	0.02	-0.27	0.99
Hefeng 50	-0.02	0.03	1.00	-0.03	0.01	0.97	-0.03	0.01	0.97	0.04	-0.36	0.97	-0.02	-0.10	1.03	0.06	-0.42	0.96
Hefeng 152	-0.02	0.02	1.00	-0.02	-0.01	1.01	0.00	-0.15	1.04	0.04	-0.42	1.04	-0.01	-0.09	1.02	0.04	-0.35	0.99
Dongnong 60	-0.03	0.07	1.00	-0.02	-0.02	1.03	0.00	-0.17	1.06	0.06	-0.48	1.04	0.02	-0.25	1.04	0.03	-0.35	1.00
Dongpu72	-0.03	0.08	0.97	-0.03	0.01	0.97	-0.02	-0.08	0.99	0.04	-0.39	0.98	0.02	-0.27	1.03	0.06	-0.46	0.97
Dongnong 253	0.00	-0.10	1.00	0.02	-0.19	0.99	0.01	-0.10	0.97	0.05	-0.39	0.97	0.03	-0.27	1.01	0.05	-0.40	0.96
Dongnong 254	-0.03	0.10	1.00	-0.02	0.02	1.01	-0.03	0.00	1.06	0.02	-0.35	1.07	0.02	-0.28	1.04	0.05	-0.42	1.03

Note:The function parameter values (a, b, c) are based on the least squares method for fitting a quadratic function ($Y=ax^2+bx+c$) to the RGR, RGE, RGI, RVI, RHL and RRL of each variety under different salt concentrations.

Supplementary Table S3. Photos of different soybean varieties under salt stress during germination

Salt Tolerant Varieties	Ctrl	100mmol/L	150mmol/L
Hei ke 58	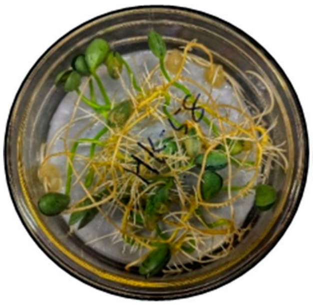	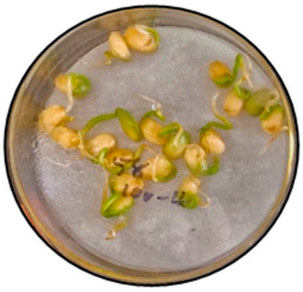	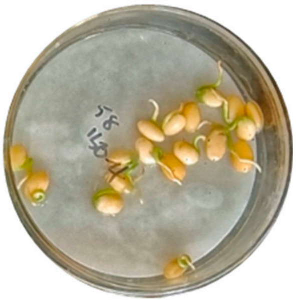
Hei ke 123	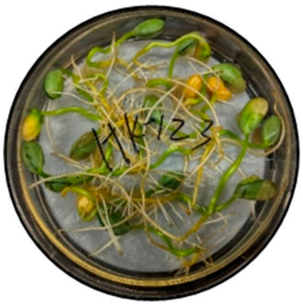	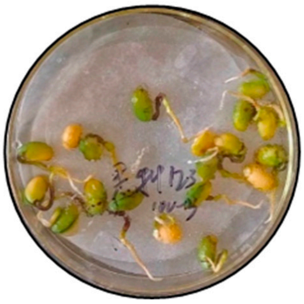	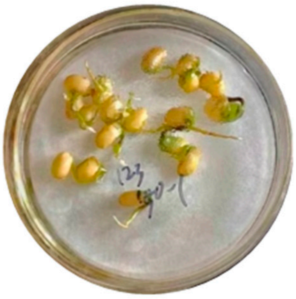
Hei ke 68	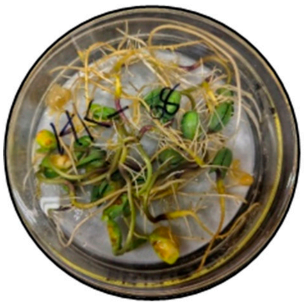	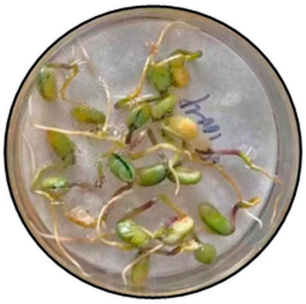	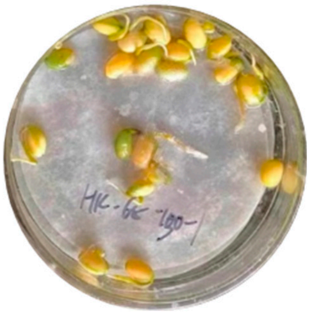
Hei he 49	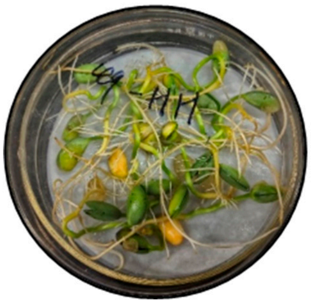	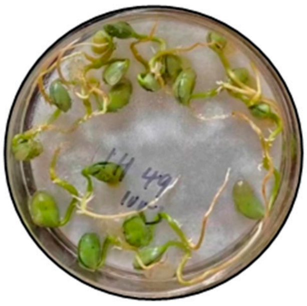	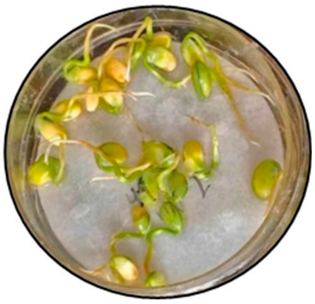
Dond nong 254	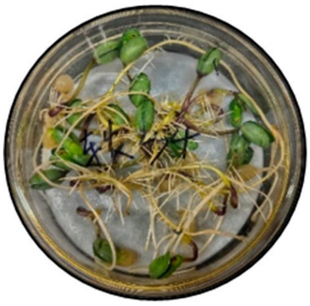	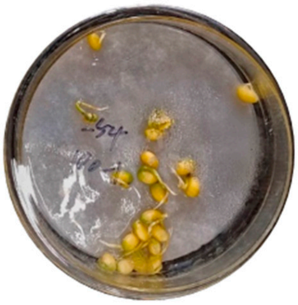	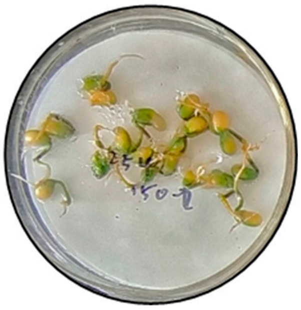

Salt sensitive varieties	control	100mmol/L	150mmol/L
Ken feng 16	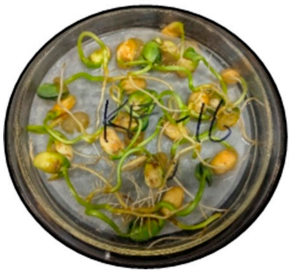	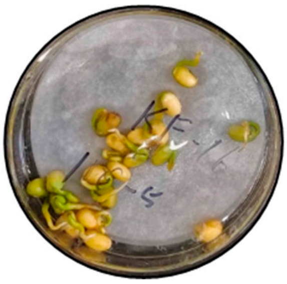	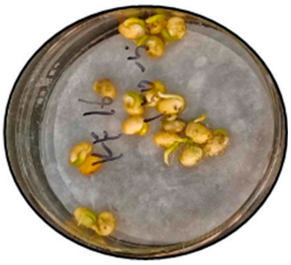
Hei nong 84	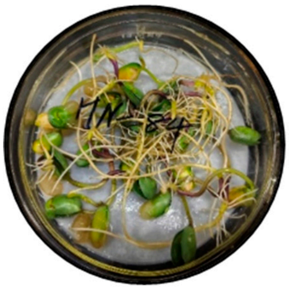	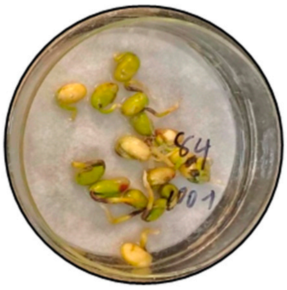	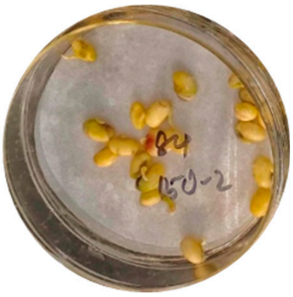
Sui nong 94	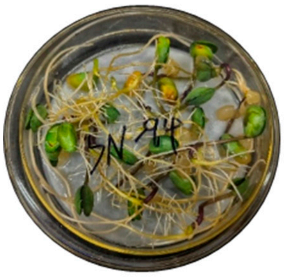	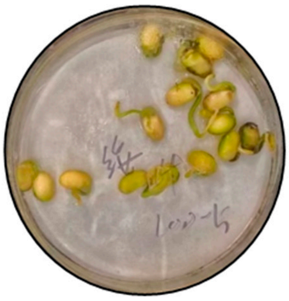	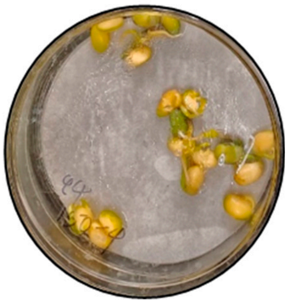

Supplementary Table S4. Acronym list

Acronym	Full name
Max	Maximum
Min	Minute
SE	Standard Error
CV	Coefficient of Variation
GR	Germination Rate
RGR	Relative Germination Rate
GE	Germination Energy
RGE	Relative Germination Energy
GI	Germination Index
RGI	Relative Germination Index
HL	Hypocotyl Length
RHL	Relative Hypocotyl Length
VI	Vigor Index
RVI	Relative Vigor Index
RL	Radicle Length
RRL	Relative Radicle Length
